# Combined Endoscopic Submucosal Dissection and Transanal Minimally Invasive Surgery for Rectal Laterally Spreading Tumor after Prior Transanal Resection: A Case Report

**DOI:** 10.70352/scrj.cr.24-0166

**Published:** 2025-05-27

**Authors:** Takashi Inoue, Fumikazu Koyama, Yosuke Iwasa, Masayuki Sho

**Affiliations:** 1 Department of Surgery, Saiseikai Chuwa Hospital, Sakurai, Nara, Japan; 2 Department of Surgery, Nara Medical University, Kashihara, Nara, Japan; 3 Department of Endoscopy, Nara Medical University Hospital, Kashihara, Nara, Japan

**Keywords:** endoscopic submucosal dissection, transanal minimally invasive surgery, rectal tumor, submucosal fibrosis

## Abstract

**INTRODUCTION:**

Endoscopic submucosal dissection (ESD) is an effective procedure for resecting noninvasive colorectal neoplasms. However, submucosal fibrosis affects the technical difficulty of ESD. We experienced a combined ESD and transanal minimally invasive surgery (TAMIS) for a rectal neoplasm with submucosal fibrosis.

**CASE PRESENTATION:**

We report our experience with a 75-year-old woman who had a rectal laterally spreading tumor with scarring from a prior transanal resection. She underwent combined ESD and TAMIS to overcome the procedural difficulty of ESD for submucosal fibrosis. The portion of the bowel without scarring was dissected using ESD, while the portion with scarring was dissected using TAMIS. A successful *en bloc* resection of the tumor was achieved, and there was no recurrence.

**CONCLUSIONS:**

Based on the findings from this case, the combination of ESD and TAMIS may be particularly effective under conditions such as rectal neoplasms with submucosal fibrosis.

## Abbreviations

EMR
: endoscopic mucosal resection
ESD
: endoscopic submucosal dissection
TAMIS
: transanal minimally invasive surgery

## INTRODUCTION

With advances in endoscopy and surgery, treatment for colorectal tumors has largely become minimally invasive. ESD is now considered feasible for the curative resection of colorectal tumors; it has a high *en bloc* resection rate and allows for detailed pathologic examination and functional preservation of organs.^1)^ Although ESD for colorectal tumors is gaining popularity, some studies have reported that severe submucosal fibrosis affects the technical difficulty of this procedure for this indication.^2–4)^ TAMIS was first reported by Atallah et al. in 2010 as a hybrid procedure involving transanal endoscopic microsurgery and single-port laparoscopy. It is a safe and feasible surgical technique that enables full-thickness excision of rectal tumors.^5,6)^ Both ESD and TAMIS are minimally invasive treatments for rectal tumors, and each has specific indications and advantages.

There has been only 1 previous case in which a pure ESD and TAMIS approach was performed for a rectal tumor with submucosal fibrosis, as our patient. In that case, *en bloc* resection of the tumor was not achieved, and local recurrence was observed.^7)^ In our case, a successful *en bloc* resection of the tumor was achieved, and there was no recurrence. This report describes the first successful *en bloc* resection of a rectal tumor with submucosal fibrosis using a pure ESD and TAMIS approach.

## CASE PRESENTATION

**Fig. 1:**
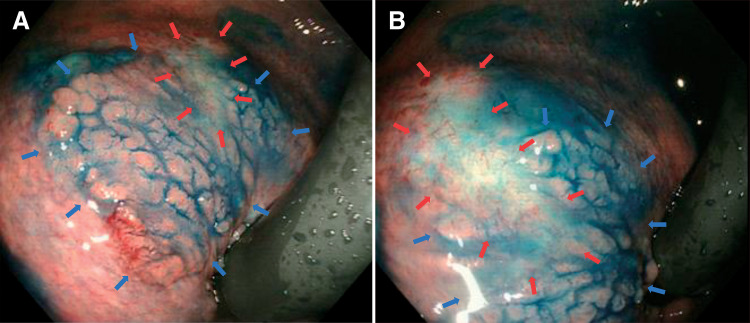
Colonoscopic findings. (**A**) A laterally spreading tumor of the lower rectum extends into the anal canal (blue arrows). (**B**) Scarring is visualized inside the lesion on the proximal side (red arrows).

A 75-year-old woman had undergone transanal resection for rectal adenoma at an outside facility. Postoperative colonoscopy showed a flat lesion at the lower rectum, and she was admitted to our department for further treatment. Colonoscopy at our institution showed a laterally spreading tumor along the anterior wall of the lower rectum, extending into the anal canal (**Fig. 1A**). The laterally spreading tumor was classified as a homogeneous type and measured about 25 × 25 mm. Scarring from her prior transanal resection was observed within the lesion on the proximal side, measuring about 8 × 6 mm (**Fig. 1B**). There were no colonoscopic findings indicative of submucosal deep invasion. Pelvic MRI could not identify enlarged lymph nodes. Because we anticipated submucosal fibrosis from the prior transanal resection, we selected a combined ESD and TAMIS approach. We planned to use ESD to dissect the portion of the bowel without scarring and TAMIS to dissect the portion of the bowel with scarring.

**Fig. 2:**
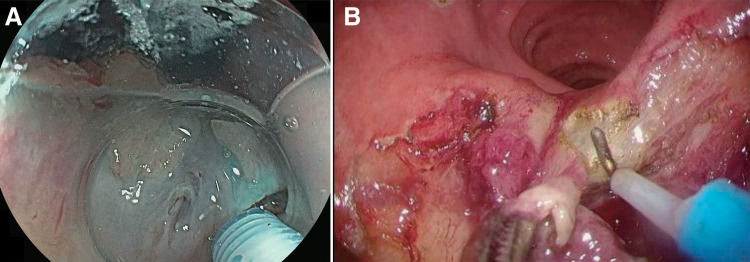
Operative findings. (**A**) Endoscopic submucosal dissection was performed distal to the tumor at the dentate line. (**B**) Transanal minimally invasive surgery was used for submucosal fibrosis at the portion of the bowel with scarring.

**Fig. 3:**
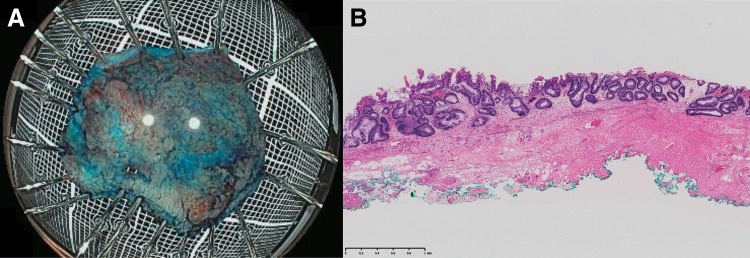
Pathological findings. (**A**) The tumor measured 25 × 23 mm and was resected *en bloc*. (**B**) Submucosal fibrosis was observed at the portion of the bowel with scarring.

General anesthesia was administered, and the patient was placed in the dorsal lithotomy position. ESD was performed using an endoscope with hood (CF-H260AZI4; Olympus, Tokyo, Japan), a flush knife (Flush Knife; Fuji Film, Tokyo, Japan), and an electrosurgical unit (VIO200D; Erbe Elektromedizin, Tübingen, Germany). We injected 0.1% adrenaline and indigo carmine locally in small doses. Diluted hyaluronic acid solution (1% hyaluronic acid [MucoUp; Johnson & Johnson, Tokyo, Japan] diluted 1.5 times with saline) was injected into the submucosa distal to the tumor. Subsequently, an incision was made into the mucosa distal to the tumor at the dentate line, and the submucosa was dissected just above the muscle layer, continuing until just before reaching the area of scarring (**Fig. 2A**). The submucosal large vessels were divided after adequate coagulation to prevent the bleeding. The mucosal incision was then extended to the same point. After ESD was completed for the portion of the bowel without scarring, there persisted submucosal fibrosis for the portion with scarring. Therefore, the portion with scarring was dissected using the TAMIS technique. TAMIS was performed using an anal retractor (Lone Star Retractor Systems; Cooper Surgical, Trumbull, CT, USA), a transanal access platform (GelPOINT Path; Applied Medical, Rancho Santa Margarita, CA, USA), an AirSeal platform (AirSeal System; CONMED, Utica, NY, USA) whose pressure was 15mmHg, and conventional laparoscopic instruments. The mucosal incision was extended proximally to make a circumferential incision. Finally, the remaining submucosal layer with scarring was dissected just above the muscle layer while applying tension with the surgeon’s left hand (**Fig. 2B**). The tumor was resected *en bloc* (**Fig. 3A**). After the tumor was removed through the transanal access device, intraluminal lavage was performed. We did not close the resected tumor site because there was no intraoperative muscle injury and she had taken no anticoagulants.

The patient’s postoperative course was uneventful. She started oral intake on postoperative day 3 and was discharged on postoperative day 5 without any complications. Histopathologic examination showed a low-grade tubular adenoma, an indeterminate horizontal margin, a negative vertical margin, and submucosal fibrosis at the portion with scarring (**Fig. 3B**). She is alive with no recurrence 3 years after the surgery.

## DISCUSSION

Because our patient had a laterally spreading tumor that was further classified as the homogeneous type,^8,9)^ she was considered a candidate for ESD. EMR was deemed difficult because the lesion extended into the anal canal. There was no significant difference in *en* *bloc* resection rates between ESD for tumors extending to the dentate line and those away from the dentate line. However, the incidence of intraoperative bleeding was 61% in patients with tumors in close proximity to the dentate line.^10)^ Therefore, we divided the submucosal large vessels after an adequate coagulation, and there was no intraoperative bleeding during ESD. Although ESD for colorectal tumors is gaining popularity, some studies have reported that severe submucosal fibrosis affects the technical difficulty of this procedure for this indication.^2–4)^ It seemed difficult to perform *en bloc* resection using the conventional ESD technique alone because of the submucosal fibrosis secondary to her prior transanal resection. Therefore, TAMIS was considered to be appropriate in this case to overcome the difficulty of dissecting the fibrosis secondary to her prior transanal resection. However, it also seemed difficult to perform *en bloc* resection using the conventional TAMIS technique alone. TAMIS has been reported as an acceptable treatment option for rectal tumors in the mid-rectum^6)^ or within 3–8 cm of the anal verge^11)^; however, the operability of TAMIS seemed to be difficult around the anal canal because of the closeness of the forceps. Additionally, ESD is more effective than transanal resection in treating noninvasive rectal tumors, with a lower recurrence rate, as the tumor margins can be clearly recognized by high-magnification chromo-colonoscopy during ESD.^12)^ Therefore, we selected a combined ESD and TAMIS approach, the portion from the dentate line up to the area of scarring was dissected using ESD, and the portion of the scarring was dissected using TAMIS, although general anesthesia was required in the operating room. There was no statistical difference in postoperative infection rates between cases where the defect following TAMIS was sutured and those left open.^13)^ Since there was no intraoperative muscle injury, we did not close the resected tumor site.

To our knowledge, there are only 3 case reports that describe the combined ESD and TAMIS approach,^7,14,15)^ and no comprehensive studies have been published. One procedure was performed for early rectal cancer with fibrosis after prior transanal resection.^7)^ Another case involved a recurrent rectal polyp after removal by EMR, where the tumor was removed using a transanal resection followed by the ESD and TAMIS approach.^14)^ Additionally, 1 procedure was conducted for a lower rectal adenoma extending above the dentate line.^15)^ There has been only 1 case in which a pure ESD and TAMIS approach was performed for a rectal tumor with submucosal fibrosis,^7)^ similar to our case. In that case, the portion of the bowel without scarring was dissected using ESD, and the portion with scarring induced by the prior transanal resection was dissected using TAMIS. Although TAMIS seemed to be effective for dissecting the portion of the bowel with scarring, the tumor was removed in 3 parts by TAMIS because of severe fibrosis, and local recurrence was observed. We also dissected the portion of the bowel without scarring using ESD and created a sufficient mucosal flap. We applied tension by grasping the flap and dissected the portion of the bowel with scarring using TAMIS. As a result, we successfully obtained an *en bloc* resection, and no local recurrence has been observed. It has been reported that piecemeal resection is a risk factor for local recurrence,^16)^ so it is important to obtain an *en bloc* resection for rectal tumors with submucosal fibrosis by using the ESD and TAMIS approaches. It may be necessary to remove a muscle layer by TAMIS if it seems difficult to perform *en bloc* resection because of severe fibrosis. This report describes the first successful *en bloc* resection of a rectal tumor with submucosal fibrosis using a pure ESD and TAMIS approach.

It has been reported that a positive horizontal resection margin after an *en bloc* ESD was associated with a marginal, nonsignificant increase in the local recurrence rate.^17)^ Although the histopathologic horizontal margin was indeterminate in our case, there was no local recurrence. Because the indeterminate horizontal margin may have been caused by damage to the specimen, improvements in gentler handling are needed.

Both ESD and TAMIS are minimally invasive treatments for rectal tumors, and each has specific indications and advantages. ESD enables the resection of tumors measuring >2 cm, an upper limit that is easily resectable *en* *bloc* by EMR, especially if the lesion extends to the submucosa. However, severe submucosal fibrosis affects the technical difficulty. TAMIS enables full-thickness resection if the lesion is located around the mid-rectum, but operability is limited around the anal canal. Therefore, the combination approach of ESD plus TAMIS enables minimally invasive treatment for rectal tumors that cannot be easily resected by ESD or TAMIS alone, as in our present case.

## CONCLUSIONS

We experienced a combined ESD and TAMIS procedure for a rectal laterally spreading tumor with scarring from a prior transanal resection. Based on the findings from this case, the combination of ESD and TAMIS may be particularly effective in conditions such as rectal neoplasms with submucosal fibrosis.

## ACKNOWLEDGMENTS

We would like to thank JAM POST (www.jamp.com) for English language editing.

## DECLARATIONS

### Funding

The authors have declared that no funding was received for this work.

### Authors’ contributions

TI performed the surgery, acquisition of data, conception and design of the work, and drafting of the work.

FK performed the conception or design of the work.

YI performed the surgery and acquisition of data.

MS critically reviewed the work and gave final approval of the version to be published.

All authors have read and approved the final manuscript.

### Availability of data and materials

De-identified patient data that support the findings of this case report are available from the corresponding author upon reasonable request.

### Ethics approval and consent to participate

This work did not require ethical considerations or approval. Informed consent for inclusion in this study was obtained from the patient.

### Consent for publication

Informed consent was obtained from the patient for the procedure and publication of this case report.

### Competing interests

The authors declare that they have no competing interests.
